# A retrospective assessment of COVID-19 model performance in the USA

**DOI:** 10.1098/rsos.220021

**Published:** 2022-10-19

**Authors:** Kyle J. Colonna, Gabriela F. Nane, Ernani F. Choma, Roger M. Cooke, John S. Evans

**Affiliations:** ^1^ Environmental Health Department, Harvard T.H. Chan School of Public Health, Harvard University, Boston, MA 02115, USA; ^2^ Department of Mathematics, Delft University of Technology, Delft 2628 XE, The Netherlands; ^3^ Resources for the Future, Washington, DC 20036, USA

**Keywords:** COVID-19, decision sciences, forecasting, uncertainty analysis, Cooke's classical model, ensemble modelling

## Abstract

Coronavirus disease 2019 (COVID-19) forecasts from over 100 models are readily available. However, little published information exists regarding the performance of their uncertainty estimates (i.e. probabilistic performance). To evaluate their probabilistic performance, we employ the classical model (CM), an established method typically used to validate expert opinion. In this analysis, we assess both the predictive and probabilistic performance of COVID-19 forecasting models during 2021. We also compare the performance of aggregated forecasts (i.e. ensembles) based on equal and CM performance-based weights to an established ensemble from the Centers for Disease Control and Prevention (CDC). Our analysis of forecasts of COVID-19 mortality from 22 individual models and three ensembles across 49 states indicates that—(i) good predictive performance does not imply good probabilistic performance, and vice versa; (ii) models often provide tight but inaccurate uncertainty estimates; (iii) most models perform worse than a naive baseline model; (iv) both the CDC and CM performance-weighted ensembles perform well; but (v) while the CDC ensemble was more informative, the CM ensemble was more statistically accurate across states. This study presents a worthwhile method for appropriately assessing the performance of probabilistic forecasts and can potentially improve both public health decision-making and COVID-19 modelling.

## 1. Introduction

The coronavirus disease 2019 (COVID-19) pandemic has had a massive toll on the world. The World Health Organization estimated that the pandemic directly and indirectly resulted in about 15 million excess deaths in 2020 and 2021 [1]. In the USA, according to the Centers for Disease Control and Prevention (CDC), more than one million cumulative deaths have been directly caused by COVID-19 [2].

Although the recent decline in rates of illness and death from the Omicron variant have suggested to some that the pandemic is behind us [3], the emergence of highly contagious subvariants, as well as the disease's apparent seasonality, has suggested that the pandemic may be with us for some time [4,5]. Public health officials and ordinary citizens have continued to seek guidance about when to mask [6,7], when to limit social activity [8,9], the importance and likely efficacy of vaccinations and boosters based on current rates of infection, disease and death [10,11], as well as projections of the likely changes in these rates over future weeks and months [12].

To provide this information, researchers have developed dozens of COVID-19 forecasting models. In the USA, since April 2020, more than 100 models have provided weekly forecasts of cases, hospitalizations and/or deaths—at the county, state and/or national level—to the University of Massachusetts Amherst's COVID-19 Forecast Hub (COVIDhub) [13,14]. These models provide not only central estimates (i.e. predictions), but also probabilistic characterizations of the uncertainty in their estimates [14].

The results have been and continue to be used to inform individual and societal decisions about how to best manage the pandemic [12,15]. Those making such decisions need to know how much confidence to place in the models' forecasts; however, very few studies have evaluated their performance. Most of what is available has focused on the performance of their central estimates (i.e. predictive performance) [16–18] and not their ability to properly characterize the uncertainty in their predictions (i.e. probabilistic performance).

Predictive accuracy is typically assessed by mean absolute error (MAE) [19] or mean absolute percentage error (MAPE) [16]. Whereas MAPE has the advantage of being scale invariant, it has been shown to favour underprediction [20]. A predictive measure based on the log of accuracy ratio [21], that considers the ratio between the predicted and true value, has been shown to overcome MAPE's drawbacks [20]. Moreover, this measure dissociates between under- and over-prediction. In this paper, we make use of the log of accuracy ratio to assess predictive performance.

One study, published recently by the team at COVIDhub, evaluates the accuracy of probabilistic incident death forecasts submitted to their data repository [19]. In their study, they measured the empirical coverage rates of prediction intervals and the probabilistic accuracy of forecasts using weighted interval scoring (WIS) [19].

This evaluation of forecast performance is incredibly useful and long overdue. However, statisticians have noted that coverage rates have drawbacks when considered in a rigorous statistical hypothesis testing framework [22]. Additionally, WIS is based on probability interval scoring (PIS). The numerical values of PIS lack direct intuitive meaning and can be difficult to interpret (please see electronic supplementary material, appendix, notes 1.3 and 1.4 for more information). Lastly, the authors evaluate model performance across a disparate set of forecasts and observations, complicating any general performance comparisons between models.

Here, in an effort to avoid these limitations, we employ Cooke's classical model (CM) for structured expert judgement [23], an established method that has been used in many studies to validate expert opinion [24–27] and that has also been suggested/implemented in other studies concerned with model forecasting [28,29]. In these circumstances, the models would serve as the ‘experts' and their forecasts would serve as their ‘judgements'. The CM also advances the idea of validation as a necessary step in aggregating experts' (here, models') uncertainty assessments, by using density-averaged performance-based weights [23]. The advantage of the CM when compared with other expert judgement methods is that it can be used to evaluate and aggregate judgements *post hoc*, without involving quantile averaging or behavioural aggregation [30–32].

In this paper, we assess both the predictive and probabilistic performance of weekly state-by-state forecasts of incident deaths provided by COVID-19 forecasting models for the most recent full calendar year (2021). A considerable effort was made to include all models which had reported sufficient data to support this performance analysis. We also compare the performance of aggregated forecasts derived from density-averaged equal and CM performance-based weights with a well-known ensemble.

## 2. Material and methods

### 2.1. Performance criteria

To evaluate the *predictive performance* of a model, for each state, *i*, and for each week, *j*, each observation, *O_i,j_*, was divided by the corresponding prediction, *P_m,i,j_*, provided by the model, *m*, to obtain the accuracy ratio, *R_m,i,j_*

$$R_{m,i,j}=\frac{O_{i,j}}{P_{m,i,j}}.$$

The distribution of the accuracy ratios was then examined. Occasionally, it is useful to discuss the bias, *B_m_*_,*i*,*j*_, of a model prediction for a specific state and week—defined as [maximum(*R_m,i,j_*(1/*R_m,i,j_*))−1].

For each model and each state, the geometric mean (GM) and geometric standard deviation (GSD) of the distributions of weekly accuracy ratios were computed and used as summary measures of that model's predictive performance in that state

$${\mathrm{accuracy}}_{m,i}={\mathrm{GM}}_{m,i}=\exp({\mathrm{mean}}_{\mathrm{over}\;\mathrm{all}\;j}(\ln(R_{m,i,j}))$$

and

$${\mathrm{precision}}_{m,i}={\mathrm{GSD}}_{m,i}=\exp(SD_{\mathrm{over}\;\mathrm{all}\;j}(\ln(R_{m,i,j})).$$

The CM was used to evaluate each model's *probabilistic performance* [23]. The CM treats these models as statistical hypotheses. It assesses ‘statistical accuracy', or ‘calibration', *C*, using the log likelihood ratio statistic, *I*_S_, which compares the forecasted probability distribution for each item with a set of corresponding observations [23,33]. Each observation may fall within one of six inter-quantile intervals, based on the five assessed quantiles (i.e. 5%, 25%, 50%, 75%, 95%) of the uncertainty distribution provided by each model forecast.

A model's calibration score, *C_m,i_*, for a state, is then

$$C_{m,i}=1\text{–}X^{2}(2\times J\times I_{Sm,i}),\;\mathrm{where}\;I_{Sm,i}=\Sigma_{k=1,\ldots ,6}\left(s_{m,i,k}\times \ln\left(\frac{s_{m,i,k}}{\;p_{k}}\right)\right),$$

where *X*^2^ is the χ^2^ distribution function with five degrees of freedom (based on the number of quantiles), *s_m,i,k_* is the fraction of the weekly observations over all weeks, *J*, in a state falling in the inter-quantile interval, *k*, provided by a model and *p_k_* is the inherent probability found in the *k*th interval [23,33]. As the divergence between *s_m,i,k_* and *p_k_* increases, *I_Sm,i_* increases and *C_m,i_* moves towards 0 (whereas the highest calibration score possible is 1). The portion 2 × *J* × *I_Sm,i_*, where *J* corresponds to the total number of assessed weeks, is the log likelihood ratio which is asymptotically χ^2^ distributed assuming the observations are independent [23,33]. *C_m,i_* is the *p*-value at which we would falsely reject the hypothesis that a model was statistically accurate for a particular state. In the context of simple hypothesis testing, it is called the significance level, *α*. At the traditional value, *α* = 5%, a model would be rejected as a statistical hypothesis if *C_m,i_* < 0.05. The CM prioritizes statistical accuracy, while informativeness serves to modulate among statistically accurate models. For more detail on the calibration score, please see electronic supplementary material, appendix, note 1.1.

Roughly, ‘informativeness’, *I*, is assessed by comparing the width of the confidence intervals provided by each model with the ‘intrinsic range' of each item [23,33]. The intrinsic range for an item is defined as the difference between the largest of the forecasted or observed values and the smallest of the forecasted or observed values [23,33]. This range is then expanded slightly by multiplying it by a user-defined ‘overshoot' factor, 1 + *F*, where *F* is typically 10% [23,33]. Using this framework, the information score for each model and each state in each week, *I_m,i,j_*, is defined as

$$I_{m,i,j}=\Sigma_{k=1,\ldots ,6}\left(\;p_{k}\times \ln\left(\frac{\;p_{k}}{r_{m,i,j,k}}\right)\right),$$

where *r_m,i,j,k_* is the probability from a uniform or log-uniform background probability density function over the intrinsic range for a model in a state for a given week in the *k*th interval [23,33]. The probability density function fit to the model quantiles is selected by the analyst in a manner that adds as little information as possible to the background measure. In our application (because the models' predictions did not exhibit high variability), a uniform background density measure was chosen. Models which concentrate their forecasts in a narrow range will have high information scores (i.e. the tighter the forecast distribution, the more information the forecasts provide). The information score also accounts for the proximity of the quantile assessments. For example, the information of an assessment increases as the 25% value is moved closer to the 50% value.

The information score for a model in a state, *I_m,i_*, is simply the average value of *I_m,i,j_* over all the weekly observations, *J*, in the state

$$I_{m,i}=\left(\frac{1}{J}\right)\Sigma_{\;j=1,\ldots ,J}(I_{m,i,j}).$$

For more detail on the information score, please see electronic supplementary material, appendix, note 1.2.

The CM combines experts' (here models’) probabilistic forecasts to form an ensemble probabilistic forecast based on the models' performances, as objectively evaluated by the calibration and information scores. As explained more fully in electronic supplementary material, appendix, note 2.2, following guidance for ‘proper scoring', the CM involves an optimization procedure to select the value of *α* which maximizes the performance score of the ensemble, *E_α_*, constructed using performance weights

$$E_{\alpha ,i,j}=\Sigma_{\mathrm{over}\;m}(w_{\alpha ,m,i}\times P_{m,i,j}),$$

where

$$w_{\alpha ,m,i}\propto C_{m,i}\times I_{m,i}\times 1_{\alpha ,m,i},$$

where *w_α,m,i_* is the normalized performance weight for a model in a state, at significance level *α, P_m,i,j_* is the forecast distribution given by a model in a state for a given week, *C_m,i_* is the calibration score of a model in a state, *I_m_*_,*i*_ is the information score for a model in a state and $1_{\alpha ,m,i}$ is an indicator variable which takes the value 1 if the calibration score for a model in a state is greater than or equal to *α*. The value of *α* is chosen to maximize the performance (i.e. *C_Eα,i_* × *I_Eα,i_*) of the ensemble. *C_m,i_* is a very fast function, typically ranging over several orders of magnitude, whereas *I_m,i_* is a slow function, typically varying by a factor 3 or less. The normalized product form of *w_α,m,i_* ensures that calibration strongly dominates over informativeness, thereby ensuring that high confidence (high *I_m,i_*) cannot compensate for low statistical accuracy (low *C_m,i_*).

Simple measures of the central tendency of a model's *national* predictive and probabilistic performance are the medians of its accuracy, precision, calibration and information scores across all states. A sense of the variability of a model's performance across states is found by examining the 5%, 25%, 75% and 95% quantiles of those distributions, as well as the median.

### 2.2. Forecast data

Weekly mortality forecasts for 2021 for each of the 50 states and the District of Columbia (DC) were gathered from COVIDhub [13,14]. This database includes forecasts of the mortality expected between one and twenty weeks ahead of the forecast date, where the weeks start on a Sunday and end on the following Saturday [14]. We focused on forecasts for weeks ending four weeks ahead and whose target end dates (i.e. the last day of the week being forecasted) occurred in 2021.

Although modellers were required to characterize the uncertainty inherent in their predictions, some did not [14]. Those who did provided quite comprehensive characterizations, typically including 23 quantiles of the probability distribution surrounding each estimate [14]. We focused on five quantiles (5%, 25%, 50%, 75% and 95%).

Occasionally, forecasts reported equal values for two or more quantiles (e.g. zero deaths at both the 5% and 25% quantiles). The method we used to evaluate probabilistic performance requires strictly increasing quantiles, which is also the requirement for the quantiles of any continuous random variable. To include these forecasts in our evaluation and at the same time remain faithful to the modeller's expressed beliefs, small, but practically insignificant, positive increasing quantities (e.g. 0.001, 0.002, …) were added to the forecasts for any adjacent quantiles which were originally identical.

### 2.3. Observation data

Observational data from a variety of sources—the Johns Hopkins Center for Systems Sciences and Engineering (CSSE) [3], the New York Times [34] and USAFacts [35]—are collected by the COVIDhub [14]. The CDC also provides observational data [36]. We relied on the Johns Hopkins CSSE data because it has been used heavily and COVIDhub has recommended that modellers use it [14].

Occasionally these databases include implausible values. When there are delays in reporting, the data repositories must retroactively adjust data, sometimes redistributing large backlogs of data to previous dates. In some instances, these adjustments led to reports of non-positive (i.e. zero or negative) deaths [3,36]. Such observations are not plausible and so any weeks with non-positive deaths have been removed from our analysis.

### 2.4. Model selection

To be included in our analysis, models must have met a set of specific inclusion criteria. Our process of model selection is illustrated in electronic supplementary material, appendix, figure S1.

One model listed in COVIDhub's data repository did not provide any forecasts and was excluded. Of the remaining 112 models which provided forecast data on 4 February 2022 [14], 30 did not provide forecasts of weekly incident COVID-19 deaths for four weeks ahead of time and were consequently excluded. Of the remaining 82 models, 16 were excluded as they did not forecast for 2021. Of the remaining 66 models, four were excluded as they provided predictions without estimates of uncertainty.

Models began forecasting at various times and many no longer provide forecasts to the COVIDhub [14]. We excluded models that did not provide forecasts for target dates spanning all months of 2021. This led to the exclusion of 35 models, leaving us with 27.

To assess model performance on the same set of observations, all models must have provided forecasts for those observations. After excluding from the analysis 29 observations where Johns Hopkins CSSE data indicated zero deaths had occurred and another seven where it indicated negative deaths had occurred, 2666 observations across 51 locations (50 states and DC) and 52 weeks remained for 2021. Eight of the 27 models provided forecasts for all 2666 observations, and if all 27 models were to be kept, the common set of observations would be restricted to 1313. These 1313 common observations included no observations in the last five weeks of 2021 and very few in the first seven weeks of the year (both periods with high mortality counts), as well as very limited coverage of Ohio and Michigan. To improve spatial and temporal coverage, two models were excluded, resulting in a final set of 1572 observations that were common to the 25 remaining models. While coverage varies by week and state, only two locations (Hawaii and DC) and 18 weeks were entirely excluded from the analysis.

Three of the remaining 25 models were ensemble models (COVIDhub_CDC-ensemble, COVIDhub-4_week_ensemble, COVIDhub-ensemble). An ensemble is simply a weighted linear combination of the outputs from individual models, and they can differ in the way weights are chosen. Common weighting schemes include equal weighting and performance weighting, using a variety of measures for performance. Because all three COVIDhub ensembles provided identical forecasts through November 2021, only one (COVIDhub_CDC-ensemble) was retained in our analysis [13,14].

**Figure 1.**
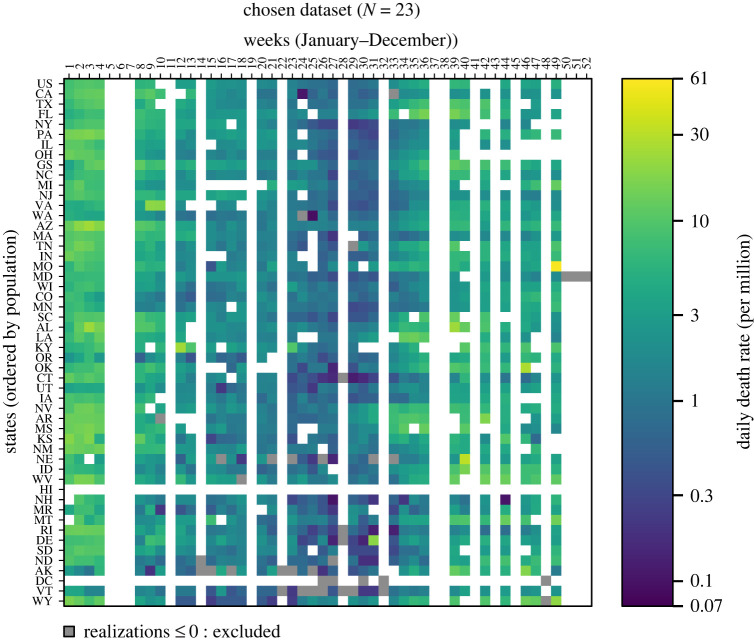
A visualization of our chosen dataset with 23 models included. Blank datapoints indicate that at least one model was missing a forecast for that forecast period and that state; these datapoints were not assessed for any model. Grey datapoints indicate that an observation was either zero or negative; these datapoints were also not assessed for any model. USA national data are shown for comparison but were not included in our dataset.

In this way, we selected 23 models—22 individual models and one ensemble. The 22 individual models considered in our analysis are: BPagano-RtDriven, CovidAnalytics-DELPHI, COVIDhub-baseline, CU-nochange, CU-scenario_low, CU-scenario_mid, CU-select, DDS-NBDS, epiforecasts-ensemble1, GT-DeepCOVID, IHME-CurveFit, JHU_CSSE-DECOM, JHUAPL-Bucky, Karlen-pypm, MIT_CritData-GBCF, MOBS-GLEAM_COVID, PSI-DRAFT, RobertWalraven-ESG, SteveMcConnell-CovidComplete, UCSD_NEU-DeepGLEAM, UMass-MechBayes and USC-SI_kJalpha. These models have been labelled with letters, from A to V, which will be used in the reporting their performance. Figure 1 provides a visualization of our final dataset (i.e. the states and weeks included), chosen in this way.

To understand whether this process led to the inclusion of models often cited in research, media and policy, a brief literature search was conducted using the Dimensions [37], Factiva [38] and ProQuest Policy [39,40] databases. The database search results, provided in electronic supplementary material, appendix, table S1, show that Model K was mentioned far more than the others in the media, and its corresponding pre-print had the highest number of citations. Although its forecasting data was limited when compared with the other models, we thought it necessary to include in our analysis, consequently limiting our potential dataset. Models G, P, I, B and L also seemed prevalent, but not nearly to the same extent as model K.

## 3. Results

### 3.1. Individual models

Of the 23 models selected from COVIDhub, 22 are individual models, and the 23rd is an ensemble (i.e. a weighted combination of the individual models). One of the individual models is a ‘baseline' model, in which predicted deaths in the target week are set equal to deaths observed in the week prior to the forecast, and the uncertainty is based on how incidence has changed from week to week in the past.

Results for the 22 individual models are reported first, followed by a comparison of the performance of the COVIDhub ensemble with two ensembles constructed using equal and CM performance-based weights. A single letter designation (from A to V) is used to indicate each model. Model C is the previously described baseline model.

The performance metrics (accuracy, precision, calibration and information) reported below reflect the medians of these measures across all states included in the analysis.

#### 3.1.1. Individual models: predictive performance

**Figure 2.**
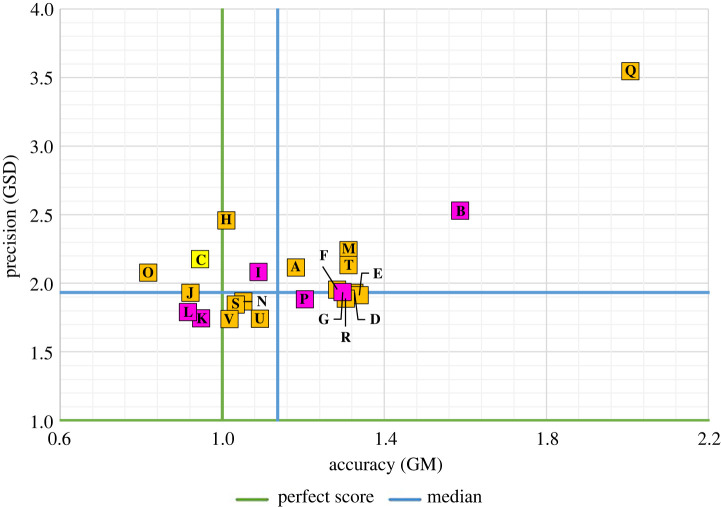
A summary of the predictive performance of the individual models. The baseline model, C, is highlighted yellow, while models that were prominent in the media are highlighted pink.

Figure 2 summarizes the predictive performance (i.e. accuracy and precision) of the individual models. Accuracy is shown on the *x*-axis, precision on the *y*-axis, and each point reflects one model.

The accuracy (i.e. the median GM of observation/prediction ratios) is between 0.9 and 1.1 for almost half of the models (10 of 22). The least accurate model (*Q*) yielded a median GM of roughly 2. The most accurate models (i.e. those in the lowest tertile of bias) are H, V, S, K, N, C and J, in order of increasing bias.

It is perhaps worth noting that although the predictive performance of most of these models is quite good, there is evidence of a tendency to slightly underestimate the observed value. The median bias across the set of 22 models evaluated is 14%. This is also visible in figure 2, as models with median GM values greater than 1 (i.e. right of the vertical green line) underestimate, on average, the weekly reported deaths.

The precision (i.e. the median GSD of observation/prediction ratios) is 1.93 or less for half of the models. The largest median GSD observed among the set of 22 models evaluated is 3.55 (model Q). All the other models have median GSDs lower than 2.55. The most precise models (i.e. those in the lowest tertile of precision) are V, U, K, L, S, N and P, in order of increasing GSD.

The models which exhibit the best predictive performance (i.e. in the lowest tertiles for both bias and precision) are V, K, S and N. For more detailed individual model results, see electronic supplementary material, appendix, table S2.

#### 3.1.2. Individual models: probabilistic performance

**Figure 3.**
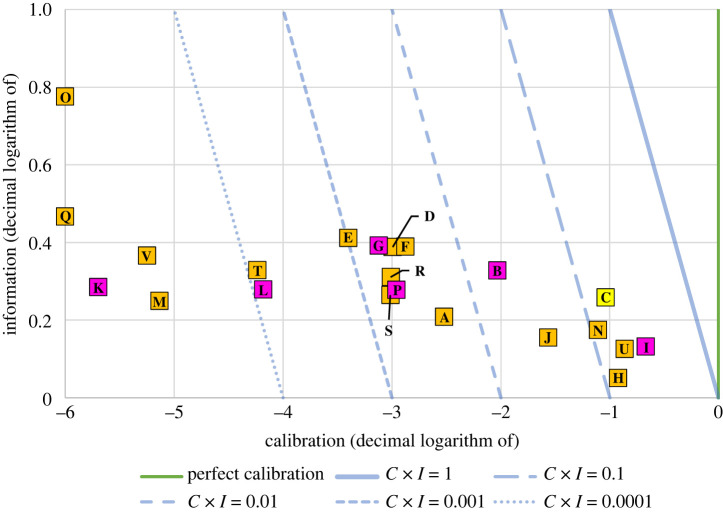
A summary of the probabilistic performance of the individual models. The light blue lines represent different isoquants of the combined score (calibration × information). The baseline model, C, is highlighted yellow, while models that were prominent in the media are highlighted pink. Although the perfect calibration score is shown (i.e. calibration = 1), there is no ‘perfect' information score; a higher information score is always preferred.

Figure 3 summarizes the probabilistic performance (i.e. calibration and information) of the individual models. Calibration is shown on the *x*-axis and information on the *y*-axis. For the figure, both values have undergone decimal logarithmic transformation.

Also shown in figure 3 are isoquants of the sum of the logarithms of calibration and information. These correspond to the products of calibration and information (i.e. the combined score), which provide the basis for the CM's evaluation of overall probabilistic performance. Increasing performance is seen as we move to the right and toward the top of the figure. The worst performing models are found in the lower left quadrant of the figure.

The models exhibiting the best probabilistic performance (i.e. in the top tertile) are I, U, C, H, N, J and B, in order of decreasing combined score. The models with the worst probabilistic performance (i.e. in the bottom tertile) are Q, O, K, V, M, L and T, in order of increasing combined score. For more detailed individual model results, see electronic supplementary material, appendix, table S2.

### 3.2. Ensembles

Of the 23 models included in our analysis, one is an ensemble—the COVIDhub_CDC-ensemble. As noted above, what differs from one ensemble to another is the approach for selecting weights and the decision of whether to weight probability densities or quantiles. During the period of interest for our analysis, the CDC ensemble relied on state- and week-specific quantiles (i.e. 5%, 25%, 50%, 75% and 95%) found by computing the medians of the values of those quantiles across all models reporting for that state and week. We were curious to know how an ensemble constructed using performance weights—based on the CM (i.e. proportional to the product of each model's calibration and information scores) and applied to densities—would perform. We also were interested to compare the performance of these two ensembles with an ensemble constructed using equal weights applied to densities (EWD).

#### 3.2.1. Ensembles: predictive performance

The predictive performance of all three ensembles was respectable—with accuracy ranging from 1.03 (CM) to 1.11 (CDC) and precision varying from 1.59 (EWD) to 2.02 (CM).

The CM-weighted ensemble was the most accurate, followed closely by the EWD ensemble. The CDC ensemble's accuracy was somewhat worse than either of the other two. By contrast, the EWD ensemble was the most precise, followed closely by the CDC ensemble. The CM-weighted ensemble's precision was somewhat worse than either of the other two. For more detailed ensemble model results, see electronic supplementary material, appendix, table S3.

#### 3.2.2. Ensembles: probabilistic performance

**Figure 4.**
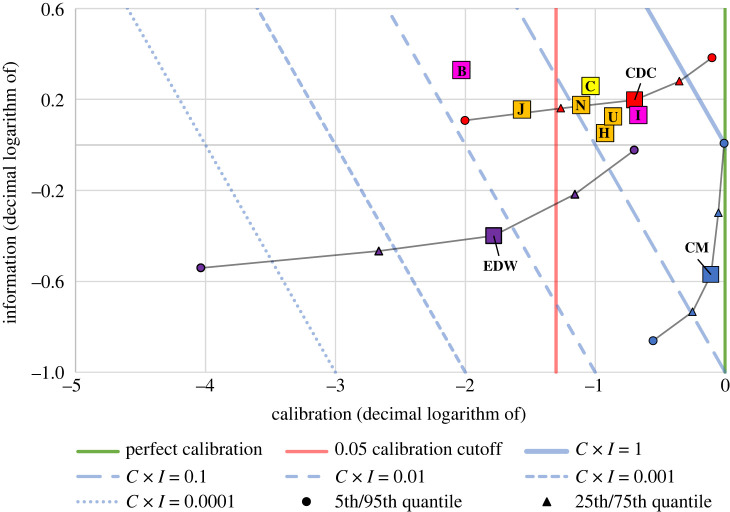
A summary of the probabilistic performance of the ensemble models, with the best probabilistic performing individual models included for comparison. The 5th, 25th, 75th and 95th quantiles of scores along with the median are shown for the ensembles. The light blue lines represent different isoquants of the combined score (calibration × information). The baseline model, C, is highlighted yellow, while models that were prominent in the media are highlighted pink. Although the perfect calibration score is shown (i.e. calibration = 1), there is no ‘perfect' information score; a higher information score is always preferred.

Large differences were observed between the probabilistic performance of either the CDC or CM-weighted ensembles and the EWD ensemble—with median combined scores of 0.32 and 0.21 observed for the CDC and CM ensembles, respectively, and a median combined score of just 0.01 for the EWD ensemble. This is apparent in figure 4, as the EWD ensemble has a much smaller median calibration score than the other two ensembles. Again, for more detailed ensemble model results, see electronic supplementary material, appendix, table S3.

Figure 4 compares the probabilistic performance of these three ensembles with the performance of the individual models found to have the best probabilistic performance (I, U, C, H, N, J and B). To provide a more complete sense of the performance of the ensembles, in addition to showing the ensemble's median performance, the figure also reflects the 5th, 25th, 75th and 95th quantile of the full distribution of each ensemble's combined scores across all 49 states included in our analysis. For more detail on these probabilistic performance scores by quantile, see electronic supplementary material, appendix, table S4.

When we examine the distribution of calibration scores across states, rather than focusing our attention on the ensembles' median calibration, the relative strength of the CM-weighted approach becomes evident. If a model were statistically accurate, one would expect it to be rejected at the 5% significance level in 2.45 of the 49 states (i.e. 5%). In this comparison, we ask, what fraction of states would the forecasts given by each ensemble be rejected as a valid statistical hypothesis at the 5% significance level? We found that, while the CM-weighted ensemble is never rejected, the CDC ensemble is rejected in 12 of the 49 states in our analysis (about 25%). The performance of the EWD ensemble is even worse; it is rejected in almost 75% of the states considered. To see these state-by-state ensemble calibration results, see electronic supplementary material, appendix, table S5.

## 4. Discussion

Amidst the ongoing pandemic, COVID-19 forecasting models have continued to play an important public health role. They have served as incredible resources for both public health officials and ordinary citizens. However, for decision-makers to understand how much confidence is warranted based on a model's forecasts, they must have access to empirical evidence on the predictive and probabilistic performance of these models.

In our analysis, the predictive performance of individual forecasting models varied substantially; however, the best models were quite accurate and relatively precise. About half of the models exhibited a median bias of less than 10%. Similarly, the median precision of more than half of the models was a factor of two or less.

The probabilistic performance of the individual models also varied substantially. Though some models exhibited decent probabilistic performance, only six of the 22 individual models had calibration scores over 0.05. This means that 16 of the 22 models were, on average, not statistically accurate across all states when *α* = 0.05. Five of the six probabilistically accurate models also had the lowest information scores. Such results clearly suggest that most of the modellers were overconfident (i.e. they substantially understated the uncertainty inherent in their forecasts).

Overconfidence is a common issue in formal elicitations of expert judgement [29,41,42]; elicitation protocols often involve elaborate procedures in an attempt to minimize this [43,44]. However, projecting future COVID-19 mortality is not easy; such efforts require an understanding of infectious disease biology and human behaviour, both of which are highly dynamic and not completely understood. To improve their probabilistic performance, modellers must better account for any limitations in their understanding of these factors when characterizing the uncertainty in their model's predictions.

It is also important to note that, of the four models (V, K, S and N) with the best predictive performance, only one (N) was among those with the best probabilistic performance (I, U, C, H, N, J and B). Models V and K, the best predictive performing models, were in the bottom one-third in terms of probabilistic performance. These results suggest that predictive performance is not indicative of probabilistic performance, and vice versa.

Surprisingly, the naive baseline model outperformed nearly all individual models. Although it was relatively imprecise, in terms of predictive accuracy, it outperformed 17 of the 22 individual models. Regarding its probabilistic performance, it had the third highest CM combined score (i.e. the product of calibration and information) of all individual models.

The ensemble models generally exhibited better and more consistent performance than the individual models. In particular, the CDC ensemble was relatively accurate, precise, and exhibited excellent probabilistic performance, on par with that of the theoretically preferred CM performance-weighted model. The large differences in information scores between the ensembles are probably due to the averaging method (quantile versus density averaging); however, quantile averaging should generally be avoided for reasons discussed previously [30,45,46] (for more detail, see electronic supplementary material, appendix, note 2.1).

The advantage of CM performance weighting becomes more evident when we examined the behaviour of these two ensembles across all 49 states included in our analysis. Here, the CDC ensemble was rejected (at the 5% level) as a valid statistical hypothesis in nearly one-fourth of the states considered, whereas the CM performance-weighted ensemble would not be rejected in any of the states.

However, it should also be noted that the CDC ensemble began using WIS-based performance weights in November of 2021. Such an ensemble would be expected to outperform the median quantile-based ensemble which they relied on during the period considered in this analysis [30,47].

Some of our results differ from the findings of Cramer *et al*. [19]. For example, where Cramer *et al*. found that models, in general, performed similarly in terms of predictive and probabilistic accuracy [19], our study found that the two measures were not indicative of each other. Similarly, Cramer *et al*. found that most of the selected forecasting models performed better than the naive baseline model [19], while our study found that most models performed worse. Their use of MAE to measure predictive accuracy and WIS to measure probabilistic accuracy, as well as their method for summarizing the scores (i.e. aggregating over all forecasts/observations and adjusting for forecast difficulty to account for their disparate dataset), probably contributed to these differences.

However, some of our findings were in agreement with those of Cramer *et al*. [19]. Both studies noted that overconfidence was a common issue among the assessed models [19]. Their results indicated that the best performing models were N, U, V, L and S [19]. Two of these models (N and U) are also on our list of top performing probabilistic models, although three (V, L and S) are not (these models did not exhibit good probabilistic accuracy; figure 3). Lastly, Cramer *et al*. found that their CDC ensemble model was consistently the most accurate model [19], and our analysis also found that this CDC ensemble performed quite well (although, it did not demonstrate the best predictive or probabilistic accuracy).

Friedman *et al*. [16], another prominent study that investigated the predictive accuracy of COVID-19 forecasting models across various global regions through February 2021, considered a smaller number of models but did include models K and V in their analysis [16]. The authors similarly found that these two models exhibited relatively good predictive accuracy [16].

There are several weaknesses to our approach. First, any interpretation of our results is complicated by the fact that forecasting models have not been static; modelling groups regularly adjust and adapt their models and have been doing so throughout the course of the pandemic. Thus, the performance assessments reported here reflect the models' average performance over the period of analysis (i.e. 2021); this report does not investigate how model performance may have changed over time. Therefore, it is important to recognize that while predictive performance depends on the models themselves, probabilistic performance is a measure of the modelling groups' ability to appropriately quantify the uncertainty in their model's predictions.

Additionally, there are other means of aggregation not evaluated here. The COVID-19 Scenario Modelling Hub has developed a particularly intriguing ensemble using a linear opinion pooling method [48]. Unfortunately, their ensemble would not meet our predefined selection criteria, as it provides forecasts infrequently and does not have forecast end dates spanning all months of 2021.

The CM is also not the only protocol that analyses and aggregates expert judgements (or, in this case, model forecasts). For example, protocols such as IDEA (investigate, discuss, estimate and aggregate) and SHELF (the Sheffield elicitation framework) rely on collective expert deliberation as a means of aggregating results (i.e. behavioural aggregation) [31,32]. Note, however, that behavioural aggregation would not be appropriate in a retrospective analysis, such as ours, because the deliberating experts would have access to past observations. Morgan and others have also raised concerns about behavioural aggregation which warrant some attention (i.e. polarized opinions, groupthink, expert dominance) [43,44].

Other weaknesses of our study include that it (i) considered only forecasts for the week ending four weeks ahead of time; (ii) considered only deaths, and not cases or hospitalizations; (iii) excluded Hawaii, DC, and some weeks, because of the limited number of forecasts or observations in those locations or weeks; and (iv) only considered forecast performance for the USA.

Conversely, our analysis has several strengths: (i) it explicitly detailed a set of specific inclusion criteria for which models must have met to be included; (ii) it evaluated the performance of numerous oft-cited and visible models; (iii) it assessed the performance of 22 individual models and three ensembles and analysed over 1500 forecasts/observations across 49 states for each included model; (iv) it examined *both* the predictive and probabilistic performance of models; and (v) it introduced a novel approach for evaluating probabilistic performance (one that has proven valuable in studies of expert elicitation).

## 5. Conclusion

In summary, we find that (i) predictive performance is not indicative of probabilistic performance; (ii) overconfidence in COVID-19 forecasting is still pervasive; (iii) most models perform worse than the naive baseline model; (iv) typically both the CDC and CM ensemble provide solid predictive and probabilistic performance; and (v) when the variability of performance across states is considered, the advantage of the performance-based CM ensemble becomes clear.

We believe our analysis demonstrates that the CM could be a useful methodology not just for formal elicitations of expert judgement, but other applications as well. The CM provides reliable and intuitive measures of probabilistic performance; avoiding the potential limitations associated with quantile averaging, WIS and any behavioural aggregation methods. We hope that users will find this approach to be valuable in their efforts to better characterize and communicate probabilistic forecast performance.

## Data Availability

Model forecasting data were gathered from the COVID-19 Forecast Hub's publicly available structured data storage repository on GitHub (https://github.com/reichlab/covid19-forecast-hub), which has been archived within the Zenodo repository: https://doi.org/10.5281/zenodo.6301718 [14]. State population data from the United States Census Bureau was required to generate figure 1 (https://www2.census.gov/programs-surveys/popest/datasets/2020-2021/state/totals/NST-EST2021-alldata.csv) [49]. Relevant code for this research work have been archived within the Zenodo repository: https://doi.org/10.5281/zenodo.6799698 [50]. All analyses were conducted in R. Data were processed and read in R v. 3.5.1 and the predictive and probabilistic performance analysis was conducted in R v. 3.6.2 [51]. The data are provided in electronic supplementary material [52].
